# Identification and quantification of macro- and microplastics on an agricultural farmland

**DOI:** 10.1038/s41598-018-36172-y

**Published:** 2018-12-18

**Authors:** Sarah Piehl, Anna Leibner, Martin G. J. Löder, Rachid Dris, Christina Bogner, Christian Laforsch

**Affiliations:** 1 Animal Ecology I and BayCEER, University of Bayreuth, 95440 Bayreuth, Germany; 2 Ecological Modelling and BayCEER, University of Bayreuth, 95440 Bayreuth, Germany

## Abstract

Microplastic contamination of aquatic ecosystems is a high priority research topic, whereas the issue on terrestrial ecosystems has been widely neglected. At the same time, terrestrial ecosystems under human influence, such as agroecosystems, are likely to be contaminated by plastic debris. However, the extent of this contamination has not been determined at present. Via Fourier transform infrared (FTIR) analysis, we quantified for the first time the macro- and microplastic contamination on an agricultural farmland in southeast Germany. We found 206 macroplastic pieces per hectare and 0.34 ± 0.36 microplastic particles per kilogram dry weight of soil. In general, polyethylene was the most common polymer type, followed by polystyrene and polypropylene. Films and fragments were the dominating categories found for microplastics, whereas predominantly films were found for macroplastics. Since we intentionally chose a study site where microplastic-containing fertilizers and agricultural plastic applications were never used, our findings report on plastic contamination on a site which only receives conventional agricultural treatment. However, the contamination is probably higher in areas where agricultural plastic applications, like greenhouses, mulch, or silage films, or plastic-containing fertilizers (sewage sludge, biowaste composts) are applied. Hence, further research on the extent of this contamination is needed with special regard to different cultivation practices.

## Introduction

Plastic debris is ubiquitous in all ecosystems on earth^1^ and yet, only a fraction of this environmental issue is visible. Through chemical, physiochemical, and biological processes, plastic debris disintegrates into smaller particles in the environment^2^. When those particles reach a size below five millimetres, they are called “microplastics”. It is commonly distinguished between fragments of larger plastic items, so called secondary microplastics, and primary microplastics which are specifically produced within a small size for a variety of applications, like cosmetic products or household cleaners. Many organisms are known to ingest microplastic particles (MPPs), with reported adverse effects from the molecular level up to the behavioural level^3^ and yet unknown consequences for environmental and human health^4^.

Research on microplastics started in the marine environment over a decade ago^5^, whereas the question of the origin of plastic debris addressed continental sources, especially rivers, as the main input route^6,7^. This led the scientific community to recently carrying out progressive work on freshwater ecosystems^8^. While both freshwater (including water surface and sediments) and atmospheric environments^9,10^ were investigated regarding microplastic contamination, there is a large gap of knowledge on the extent to which terrestrial ecosystems are affected^1^. This is even more surprising, as plastic is already suggested as a stratigraphic indicator for the Anthropocene Era due to its suspected omnipresence in soils^11^. Recent literature^8,12–14^ illustrated several pathways for plastic debris entering terrestrial soils. In this context, Hurley and Nizzetto^14^ differentiated between three main categories of sources: (I) fragmentation of plastic debris already present in the environment, (II) deposition and runoff from the surroundings, and (III) inputs from agricultural practices. Thereby, fragmentation of intentional or unintentional discarded plastic debris is one of the major input routes, with estimates that around 32% of the world plastic packaging debris can escape collection systems^15^. These macroplastics further represents a constant input source for secondary microplastics to the environment if not removed. In addition, aeolian transport constitutes a possible distribution route for small microplastics from their source to remote areas where deposition to soils (due to rain, declining wind, or barriers) could occur^10,16^. Runoff from the surrounding environment is also a potential pathway, but its influence is yet unknown. While it is widely discussed for microplastics^17^, field studies quantifying it are lacking^13^. Next to those sources, it has recently been suggested that fertilizers originating from sewage sludge^18^ and bio-waste^19^ processed from households or commercial bindings, may act as a further major input route of microplastics to soils and thus, especially agricultural soils are prone to microplastic contamination. For instance it is assumed that between 63,000 and 430,000 tons of microplastics are added via sewage sludge to European farmlands annually^18^. Nevertheless, the main source of nitrogen input to agricultural lands within the EU is mineral fertilizer and livestock manure, accounting for 83.2%^20^. Consequently, the question arises if also a conventionally treated agricultural farmland not subjected to potentially microplastic-containing fertilizers, could still be contaminated with plastic debris.

We therefore collected data on the amount of macro- and microplastic debris on an agricultural farmland in southeast Germany. We chose a study site which only receives conventional agricultural treatment (rainfed, ploughing, harrowing, sowing, fertilization, herbicide application, harvesting) and is not subjected to microplastic-containing fertilizers (for example sewage sludge, organic fertilizers) or agricultural plastic applications (for example mulching films, greenhouses, nets). Therefore, our study is the first report on macro- and microplastic contamination of an agricultural farmland in rural areas which only receives conventional agricultural treatment.

## Results

**Figure 1:**
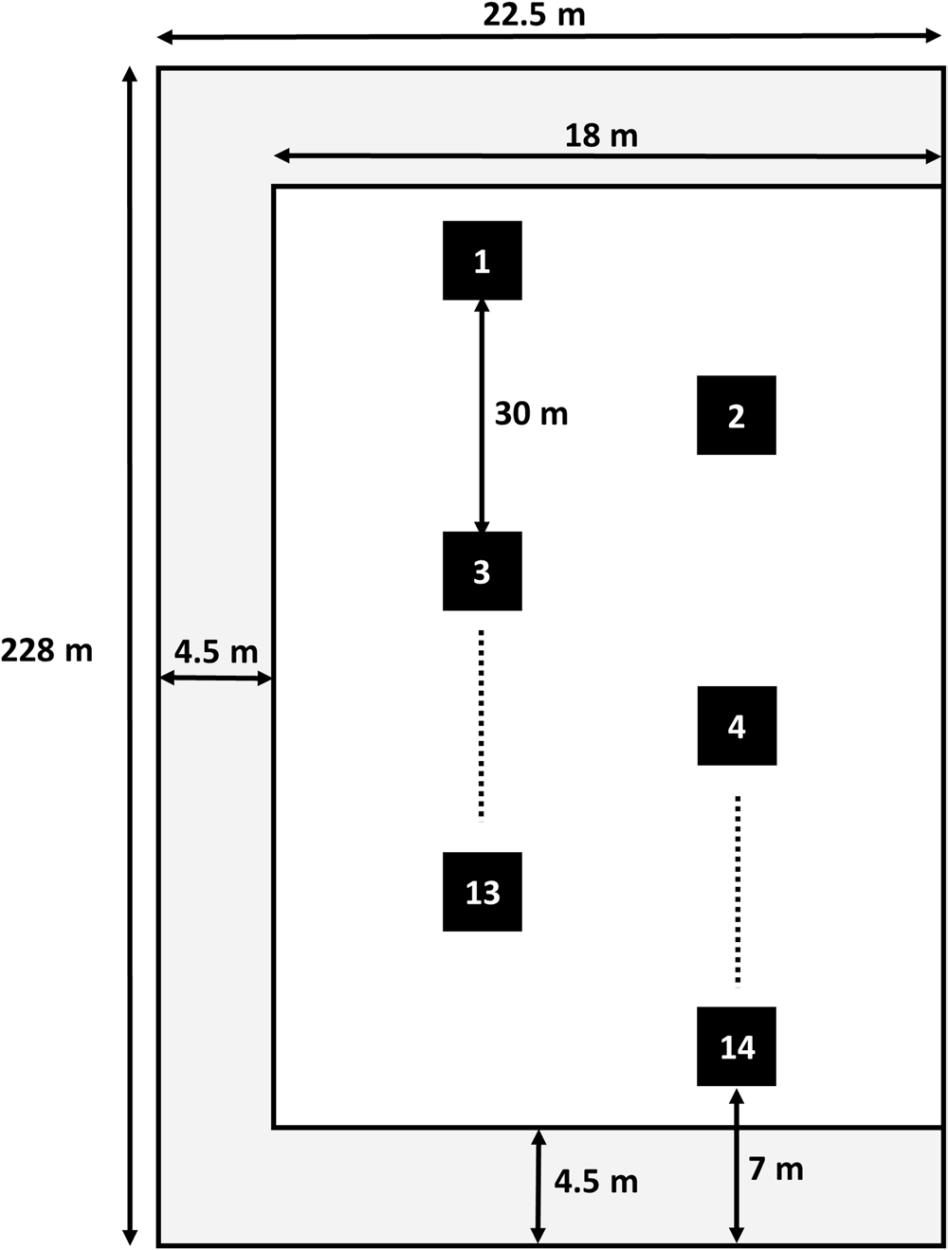
Schematic drawing of the investigated agricultural farmland with length specifications and placing of the samples for microplastic analysis (1 to 5 mm). The margin is shown in grey, the sampled area for macroplastics in white. On the site bordering another similar cultivated agricultural field, no margin was applied. Black dots indicate further sampling plots not shown in the figure.

An agricultural farmland in Middle Franconia, southeast Germany with a total area of 0.5 ha (Fig. 1) served as the study site. Geographically, it can be assigned to the supra-region “Southwest German Scarplands”. This is a wide flat open cultural landscape shaped by arable land. The exact coordinates of the field cannot be provided in order to respect the privacy of the owner. The soils in this area are predominantly Entisols and Vertisols (both USDA soil taxonomy and FAO WRB) with a high clay content^21^. The site is located approximately 316 m above mean sea level and has a slope of ~6%, facing north-northwest. It is bordered by field roads on three sides and surrounded by fields owned by the same person (i.e. receiving similar agricultural treatment). The closest inhabited area is located 1.5 km distance away and the next bigger city of Nuremberg around 50 km. Within the last five years, the investigated field was fertilized with pig and cow manure as well as ammonium sulphate nitrate fertilizer. No agricultural plastic was used for cultivation practices on the field and cultivated crops include wheat, barley, lucerne, triticale, white mustard, and corn. The farmland was regularly ploughed to 20 to 30 cm depth. Barley was sowed two weeks prior to our sampling and at sampling seedlings had reached a height of around 5 to 10 cm. To exclude an influence from the surroundings, a margin of 4.5 m was left out along three sides adjacent to a field road during sampling, according to the guidelines for sampling of arable soil^22^, thus a total area of 0.4 ha was analysed. Macroplastic debris was collected from the soil surface, whereas for microplastics (1 to 5 mm), 14 samples were taken from the top 5 cm of soil within 32 × 32 cm quadrates, resulting in a sample volume of approximately 5 litres per sample (see Methods).

### Macroplastic contamination

**Figure 2:**
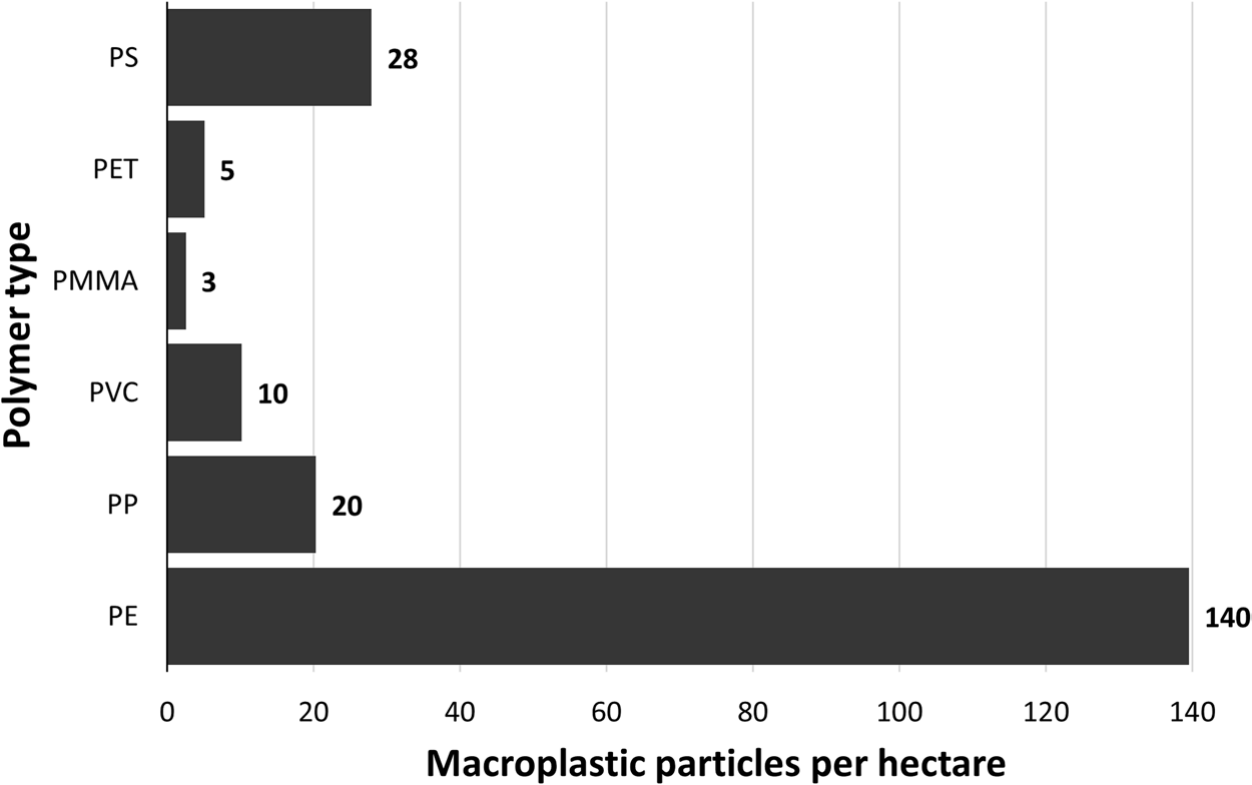
Abundance of polymer types within the detected macroplastic particles on the investigated agricultural farmland. Particles are separated by polymer type on the y-axis and show particle numbers per hectare at the end of each bar. Polymer types are sorted by polymer application amounts for European agriculture (no data for PET and PS)^28^, which decrease from bottom to the top of the y-axis. PE, polyethylene; PP, polypropylene; PVC, polyvinyl chloride; PMMA, polymethyl methacrylate; PET, polyethylene terephthalate; PS, polystyrene.

**Figure 3:**
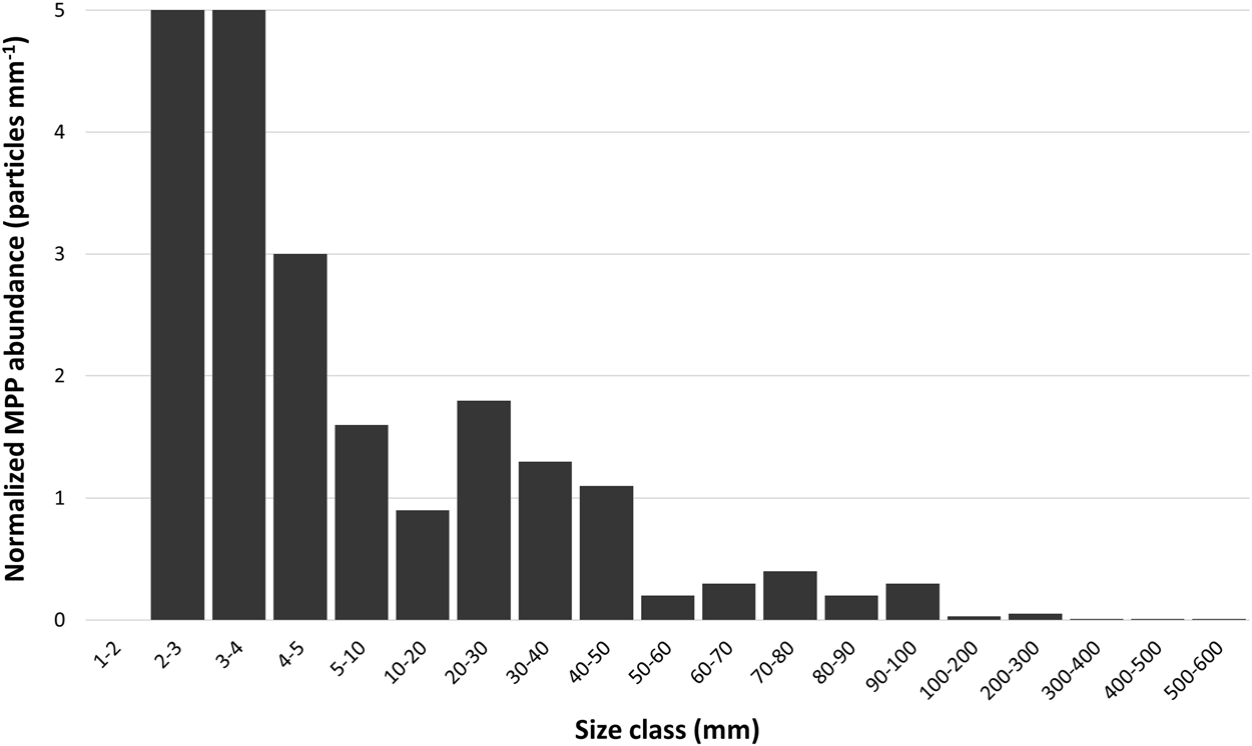
Size distribution of plastic debris detected on the investigated agricultural farmland. Normalized particle abundances (particle abundance of a size class divided by the width of that size class in millimetre) are shown on the y-axis and size classes in millimetre on the x-axis. Particle length was used to sort particles into size classes.

**Figure 4:**
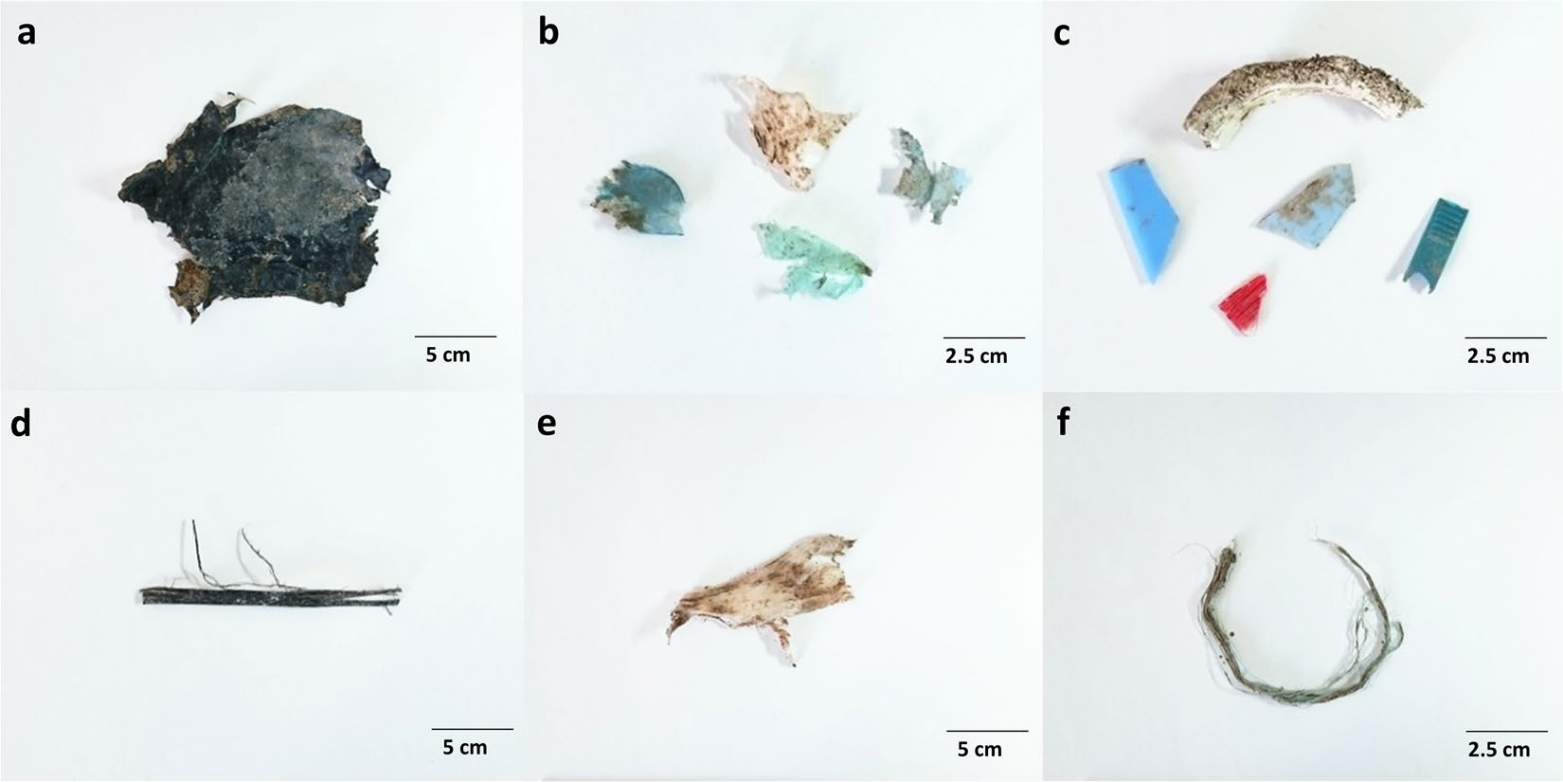
Exemplary pictures of macroplastic debris detected on the investigated agricultural farmland. Particles were grouped into three different shape categories: films (**a**,**b**), fragments (**c**), and others (**d**–**f**).

**Figure 5:**
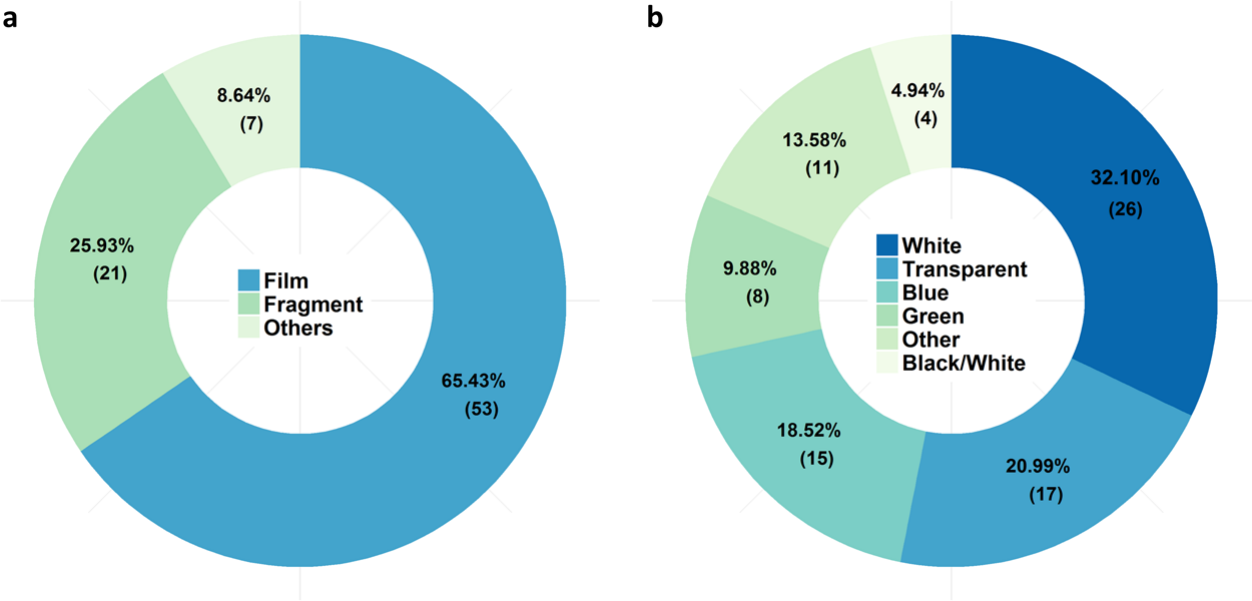
Relative proportions of shape (**a**) and colour (**b**) of macroplastic particles found on the investigated agricultural farmland with absolute numbers in brackets. Black/White refers to bicoloured films, featuring a black and a white side.

Using attenuated total reflection (ATR)-Fourier transform infrared (FTIR) spectroscopy we identified 81 macroplastic pieces collected within the investigated area which extrapolates to 206 particles per hectare. In total, six different plastic polymers were identified. The most common polymer type found was polyethylene (PE) with 67.90% (55 particles) of all macroplastic pieces, followed by polystyrene (PS; 13.58%, 11 particles) and polypropylene (PP; 9.88%, 8 particles). Pieces of polyvinyl chloride (PVC; 4.94%, 4 particles), polyethylene terephthalate (PET; 2.47%, 2 particles), and polymethyl methacrylate (PMMA; 1.24%, 1 particle) were also found (Fig. 2). Size distribution analysis revealed that over two-thirds (72.84%) of all macroplastic pieces were in the size range of 5 to 50 mm and only three pieces >300 mm were found (Fig. 3). The calculated total surface area of the found macroplastic pieces corresponds to approximately 0.18 m^2^. The measured total weight added up to 26.25 grams. As for the number of particles, PE is also predominant in terms of mass with 16.39 grams (62.43%), followed by PS (15.80%, 4.15 grams), PP (8.57%, 2.25 grams), PVC (7.99%, 2.10 grams), PET (3.88%, 1.02 grams), and PMMA (1.34%, 0.35 grams). Most of the found plastic debris were in the shape of films (65.43%), which together with fragments (25.93%) comprised 91.36% of all found macroplastic pieces (Figs 4 and 5A). Other shapes like rope, strapping tape, and fabric rests were grouped together in the category “others” with a 8.64% contribution. The found colours were more diverse, with white (32.10%), transparent (20.99%), and blue (18.52%) being the most common ones (Figs 4 and 5B), followed by green and bicoloured particles which often featured a black and a white side.

### Microplastic contamination

In total, 16 MPPs (5 to 1 mm) were identified via ATR-FTIR spectroscopy in ~50 kg dry weight (DW) of the sampled soil. Particle abundances in samples ranged from 0 to 1.25 MPPs per kilogram DW with a mean abundance of 0.34 ± 0.36 MPPs per kilogram DW (Table 1). Using the typical bulk densities for clayey soils (between 0.93 and 1.72 g cm^−3^ ^23^) and considering the sampled upper 5 cm of soil we can transform the average abundance of MPPs per weight (0.34 MPPs per kilogram DW) to the average abundance of MPPs per area. Thus, we can estimate that the soil contains between 158,100 and 292,400 MPPs per hectare. This must be seen as a rough extrapolation, considering the high standard deviation of the average abundance of MPPs.

**Table 1:** Microplastic particle (MPP) abundances in absolute numbers of polymer type found within the samples and total MPP abundances per sample and per kilogram dry weight. PE, polyethylene; PP, polypropylene; PS, polystyrene.

Sample ID	Polymer type			MPPs per sample	Weight of sample in kilogram	MPPs per kilogram
	PE	PP	PS			
Plot 01	0	0	0	0	3.7991	0
Plot 02	1	3	0	4	3.2099	1.2461
Plot 03	1	0	1	2	3.4249	0.5840
Plot 04	1	1	0	2	3.5579	0.5621
Plot 05	1	0	0	1	2.9504	0.3389
Plot 06	0	0	0	0	3.3204	0
Plot 07	1	0	0	1	3.2635	0.3064
Plot 08	1	0	0	1	3.2539	0.3073
Plot 09	2	0	0	2	3.6726	0.5446
Plot 10	2	0	0	2	2.9978	0.6672
Plot 11	0	0	1	1	4.3150	0.2317
Plot 12	0	0	0	0	4.7340	0
Plot 13	0	0	0	0	3.4402	0
Plot 14	0	0	0	0	3.4414	0
Mean	0.7143	0.2857	0.1429	1.1429	3.5272	0.3420
SD	0.7263	0.8254	0.3631	1.1673	0.4883	0.3585

**Figure 6:**
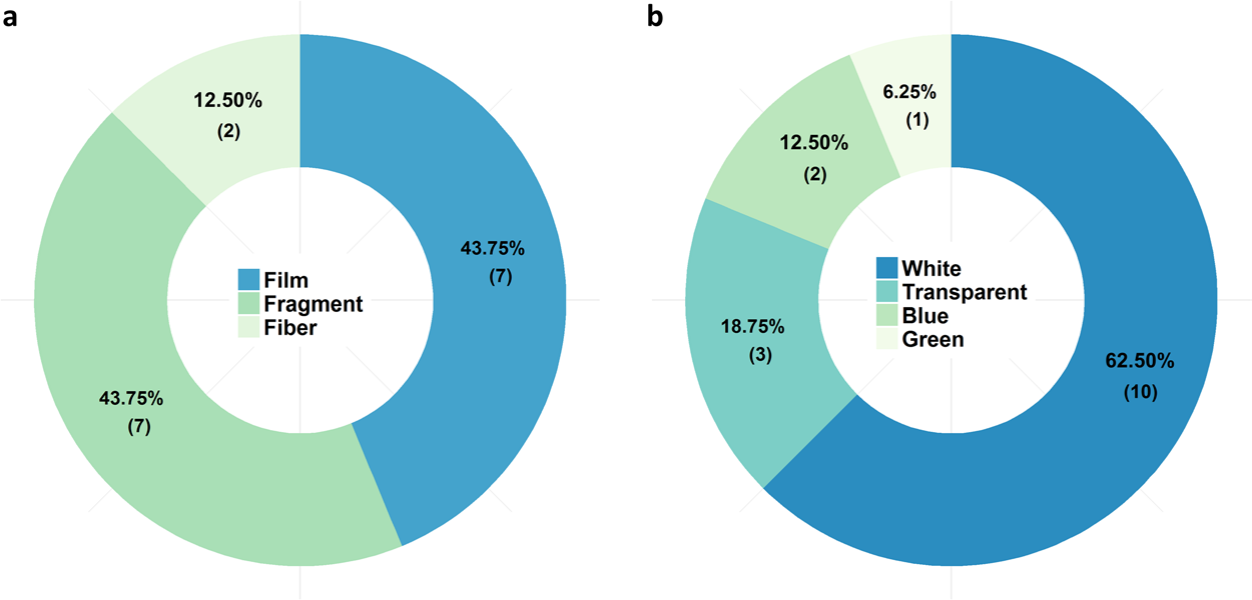
Relative proportions of shape (**a**) and colour (**b**) of microplastic particles found on the investigated agricultural farmland with absolute numbers in brackets.

Polyethylene was the polymer type most often found (62.50%, 10 particles), followed by PP (25.00%, 4 particles), and PS (12.50%, 2 particles) (Table 1). The total weight of the found MPPs added up to 0.005 grams. With 43.75% each, fragments and films were the most common shapes of MPPs (Fig. 6A). Only two polymer fibres (12.50%) were identified within the samples (Fig. 6A). The majority of MPPs were white (62.50%). Only a few MPPs with a transparent, blue, or green colour were found (Fig. 6B). According to size measurements, no particles below 2 mm length were found within the samples (Fig. 3). Two fibres and one particle slightly longer than 5 mm were included in the microplastic fraction as they passed the mesh size of 5 mm. No plastic particles were detected in the negative control.

## Discussion

### Macroplastic contamination

In our study we found a concentration of 206 macroplastic pieces per hectare. Although, the site was screened by two independent observers for macroplastic debris, we cannot completely rule out that some macroplastic pieces, especially when covered with soil, were overlooked. Thus, our reported number is rather conservative. To our best knowledge, there are only a few quantitative studies on macroplastic debris in terrestrial ecosystems and no full quantitative study for agroecosystems. This lack of data limits the possibilities to interpret and compare the results of the present study. Zylstra^24^ examined an area within the Sonoran Desert National Park, southern Arizona, which is relatively unaffected by human littering but still found macroplastic densities ranging between 0.056 and 0.627 pieces per hectare. However, they only focused on plastic bags and balloons. In contrast, Basnet^25^ selected a hotspot within the Sagarmatha (Mt. Everest) National Park and counted solid waste on tourist trekking trails as well as camp disposal sites. They detected 0 to 1,769,870 macroplastic pieces per hectare with a mean value of 141,190.56 ± 372,651.96 pieces per hectare. A similar order of magnitude for plastic debris was found by Huerta Lwanga *et al*.^26^. They focused on house waste disposal sites (50 × 50 m) within home gardens in a rural environment in Mexico and quantified 744,000 ± 204,000 PE bottles and 74,000 ± 65,000 plastic pieces per hectare. Our results lie between those reported numbers and reflects contamination in a region with intensive agricultural land use and proximity to rural communities. Besides, investigations of arable lands with long-term plastic film mulch covers revealed soil residual plastic mulch levels from 50 to 260 kilograms per hectare^27^, primarily composed of PVC. The calculated weight of the found macroplastic debris within our study amounts only to 0.066 kilogram per hectare and is composed of more diverse polymer types. This indicates, that plastic contamination is to a considerable degree higher on farmland where plastic materials are applied on a regular basis, compared to farmland where no agricultural plastic is used for cultivation practices.

Besides PET the identified polymer types are among the most commonly used polymers in agriculture, such as PE, followed by PP, PVC, ethylene vinyl acetate (EVA), PMMA, and polycarbonate (PC)^28^. Nevertheless, only few particles can be linked to agricultural origin, such as bicoloured fragments of black and white (4.94%), which may originate from so called white-on-black mulches or silage films that are frequently used in agriculture^29^. Even less common were distinct particles like an ear tag from livestock or an animal feed packaging fragment. It is possible that the applied pig and cow manure contained plastic debris, as plastic materials are omnipresent at farms, and unintentional breakdown of materials and spreading may introduce plastic debris into farmyard manures. Furthermore, plastic debris unintentionally attached to agricultural equipment (i.e. tractors), as for example white-on-black films, may be transferred to the agricultural soils. As all neighbouring farmlands are owned by the same person, receiving the same agricultural treatments, transfer of plastic debris from those farmlands is probably a minor route. In addition, careless disposal and displacement of plastic by wind were supposed to be the major routes for input to the national park in the Sonoran Desert^24^ and are further possible pathways relevant for this study.

### Microplastic contamination

The found mean abundance of 0.34 ± 0.36 MPPs per kilogram DW is a conservative measurement as we cannot completely rule out that microplastic particles could have been overlooked during visually pre-sorting for subsequent ATR analysis. Nevertheless, visual pre-sorting has been suggested for larger particles^30,31^ and is commonly applied^32^. It further needs to be considered that the found numbers represent only a part of the whole microplastic contamination due to the applied lower size limit of 1 mm.

The presence of microplastics in agricultural soils was already suggested by a study that investigated the potential to use synthetic fibres as an indicator of sewage sludge application^33^. The study considered all fibres as synthetic if displaying certain characteristics under a polarized light microscope but did not identify the chemical nature of these fibres. However, pure visual identification of synthetic materials is often error-prone since particles of natural origin can have the same appearance as synthetic fragments^6,8^. Hence, the present study used FTIR spectroscopy to quantify the presence of plastic particles in soils. Fibres accounted only for 12.50% of the microplastics within this study, indicating that all shapes of microplastics have to be considered if analysing the microplastic contamination on farmlands. The low amount of fibres could be related to the fact that on the studied site no sewage sludge was deployed as fertilizer. A more recent study investigated two different soil horizons but only PE and PP were considered^34^. Both polymer types were identified based on an observation of the melting products before/after heating the samples at 130 °C for 3–5 seconds. As this is a new method which cannot address the chemical nature of the object under consideration, it still requires further validation and its efficiency needs to be determined. The study suggests that PE and PP particles down to <50 µm in size were present in an agricultural soil where plastic mulch was applied for at least 20 years. In the present study, plastic types were chemically identified and a wider variety of plastic materials have been found. The fact that plastic mulch was not used in the studied farmland shows the existence of other potential microplastic sources for agricultural fields.

Input via runoff from adjacent areas is unlikely at our study site, as the field is located on top of a hill. Additionally, the drainage systems surrounding the study site exhibit another barrier for input via runoff from adjacent areas. Atmospheric deposition of microplastics was shown in a study from Dris *et al*.^9^, predominantly finding fibres in the size range of 200 to 600 µm in the atmospheric fallout of urban and sub-urban sites. Regarding particles, atmospheric suspension of quartz grains occurs at a particle size <70 µm^35^. Atmospheric transport of the investigated polymers >1 mm (densities of 0.9 to 1.4 g cm^−3^) seems therefore rather unlikely, even if considering the higher density of quartz (2.65 g cm^−3^). However, we cannot rule out that transport way completely. Regarding particle motion processes, only pushing or rolling over the surface (so called “surface creep”) could be an aeolian process accounting for microplastic transport in the investigated size class.

Another apparent source for the found MPPs in this study could be the onsite degradation of macroplastic debris on the field. This assumption is supported by our results: PE films were most often identified for macroplastics (56.10%, 46 particles) and concordantly most of the identified microplastics were PE films (43.75%, 7 particles) and fragments (18.75%, 3 particles). Moreover, this is reflected by the identified colours: 65.22% of the macroplastic PE films where transparent (17 particles) or white (13 particles); in accordance 50.00% of the microplastic PE films and fragments were transparent (3 particles) or white (5 particles). For example, ploughing could entrap macroplastic debris at the soil surface, thereby increasing its residence time on the field. Partly trapped macroplastics at the soil surface are directly exposed to UV light, initiating photo-degradation which is recognized as a major process for decomposition of polymer materials^36,37^. If already brittle, further mechanical disintegration, caused by application of shearing forces, can occur through several mechanisms like freeze-thaw-cycles, pressure due to burial under soil or snow, or damage caused by interactions with organisms^38,39^. Further, shearing forces on plastic debris in agricultural soils could act when the farmland is ploughed and other tillage work is done. If macroplastics are present on the agricultural farmland, all the processes described above will lead to a constant production of new microplastics through fragmentation.

Rough estimations of plastic debris abundances within the upper 5 cm of soil further suggest that MPPs (1 to 5 mm – 158,100 to 292,400 particles per hectare) are several orders of magnitude more abundant than macroplastics (>5 mm – 276 to 510 pieces per hectare if similar approximations are made for the upper 5 cm, backed up by observations that several pieces were partly buried within the soil). If transferring the particle numbers to mass, macroplastics were more abundant with 0.066 kilogram per hectare, compared to 0.035 kilogram microplastics per hectare, considering only the sampled surface area for microplastics. Moreover, the size distribution of the found plastic debris is left-skewed (increasing abundances with decreasing size, see Fig. 3), which can be explained by progressive fragmentation of larger pieces into more and smaller pieces^40^. Understanding of particle size distribution of MPPs is still limited due to a lack of comparable data. It is assumed that particle abundance is increasing logarithmically with decreasing particle size as already observed for natural particles^32^.

Further studies are necessary to understand transportation processes of plastic debris on soil surfaces and within soils to determine the fate and possible sinks for plastic debris. Microplastics present at the soil surface presumably are transported by wind or are transferred into surface waters by run off after rain events^17^. Only two studies have demonstrated that microplastics could be transported along the soil profile by earthworms and via preferential flow paths^41,42^. Soil structure of agroecosystems is very dynamic in the ploughed horizon and macroporosity, necessary for preferential flow, is regularly destroyed by tillage. However, the lower soil below the tillage pan could still be hydraulically connected to the upper soil, in particular in clayey soils that tend to shrink when drying^43^. Thus, at sites with a shallow groundwater and soils with a shrink-swell potential, for example, a downward transport of microplastics to the groundwater could be possible.

## Conclusions

The ubiquitous contamination of the environment with microplastics has recently attracted a great deal of public and scientific attention. Surprisingly, only little is known for terrestrial ecosystems although it is a crucial missing link in the global understanding of plastic distribution in the environment. This study presents first quantitative and qualitative data of plastic contamination in an agricultural soil which only receives conventional agricultural treatment. It therefore provides important data on microplastic contamination for agricultural areas where microplastic-containing fertilizers and agricultural plastic applications are not used. However, our results are probably at the lower end of possible contamination levels in agricultural farmlands, as much higher levels can be expected in areas where plastic application is common^44^. Since agricultural land covers more than one third of the global land area^45^, this issue should be treated with the same global importance as marine and freshwater ecosystems. Whether the presence of plastic debris in agricultural soils has implications for economy and environmental health is already under discussion. For instance, studies on residual plastic mulch levels within agricultural soils report negative effects on crop emergence, root growth, and salinization^27^. Further effects on moisture and nutrient transport in soils are hypothesized^27^. Hence, further research on agroecosystems and other terrestrial ecosystems will shed light on the significance of this contamination on land and pave the way to rethink the current paradigm that the oceans are the only sink for plastic debris.

## Methods

### Macroplastic sampling

The field survey was conducted on 13th October 2015. To account for potential influences from the adjacent field roads (through farm tractors or pedestrians), we split the study site into two areas: margin and centre area (Fig. 1). A minimum distance of 4.5 m from the edge of the field was chosen to account for any influences of the surroundings. Macroplastic debris (all visible particles >0.5 cm) was collected by two persons as follows: the total area was divided into 13 transects with a distance of one arm length (approximately 80 cm) in between. Each transect was walked by one observer, whereas the distance between transects allowed observation of the whole area by both observers. All potential plastic fragments were collected from the soil surface (pieces per hectare) and transferred into freezer bags for further analysis in the laboratory. Macroplastic particles were then photographed (Canon Digital Ixus 50), measured, and analysed via ATR-FTIR (attenuated total reflectance – Fourier transform infrared) spectroscopy as described below.

### Microplastic sampling

Microplastic sampling was performed on the same day as the macroplastic sampling and according to the protocol developed by the MSFD Technical Subgroup on Marine Litter with some adaptations^46^. We took 14 evenly distributed samples aligned in two transects within the centre area of the farmland (Fig. 1). Each sample was taken from a 32 × 32 cm quadrate made of stainless steel and the upper 5 cm of soil extracted. Thus, at each sampling plot around five litres of soil were transferred into PE barrels using metal spatula. To account for possible contamination by the PE barrels, we prepared one negative control by adding de-ionized water to the barrel and treated it like all other samples.

To normalize microplastic abundance to kilogram soil, the dry weight of each sample was first determined. To do so, the wet weight of a subsample (100 to 200 g) from each sample was determined on a laboratory scale (Sartorius 1219 MP), followed by drying (at 60 °C) until a stable weight was reached. Afterwards each subsample was analysed for microplastics as described below.

### Microplastic sample purification

A challenge in any microplastic study is to overcome the problem of very heterogenous sample matrices which interfere with the MPP detection. Considering both, the highly labour-intensive analysis of particles <1 mm and the large area of the investigated site we decided on taking large sample volumes and thus to focus on particles >1 mm. As we concentrated on MPPs with a size range of 1 to 5 mm, we first conducted a volume reducing step where all material smaller than one millimetre was discarded.

A simple wet-sieving of the clayey soil was not possible, as soil aggregates could not be destroyed despite soaking samples over several days with de-ionized water. Therefore, samples were transferred into glass beakers and soil aggregates were dissolved as follows: 500 ml of sample was covered with de-ionized water and 20 ml of hydrogen peroxide was added. After the reaction (formation of bubbles, gasification) stopped, size fractioning was done with two combined sieves with a mesh size of five and one millimetre respectively. Those steps were repeated until all soil aggregates were dissolved (maximum of three times). The residual potential MPPs on the sieves were optically sorted under a magnifying lamp, further viewed under a stereomicroscope (Leica M50), photographed (Olympus, DP 26), and stored in Eppendorf tubes for subsequent ATR-FTIR analysis.

### Analysis

For identification and quantification, all potential plastic particles were analysed with a Tensor 27 FTIR spectrometer (Bruker Optik GmbH) further equipped with a Platinum-ATR-unit (Bruker Optik GmbH). For the measurement of each particle, 16 background scans were pooled, followed by 16 sample scans with a spectral resolution of 8 cm^−1^ in a wavenumber range from 4000 cm^−1^ to 400 cm^−1^. Obtained spectra were analysed with the software OPUS 7.5 (Bruker Optik GmbH) through comparison with polymer reference spectra from a custom-made database containing the most common polymers as well as natural materials^47^. Besides polymer type, colour and shape of each particle were recorded. Measurements of macroplastic area and length measurements for micro- and macroplastic were conducted with the image processing program ImageJ (version 1.51j8). If macroplastic pieces exhibited holes, those areas were subtracted from the total area. As the width of the size classes for particle size distribution analyses were not uniform, the particle numbers were normalized by dividing the total particle numbers of each size class by the width of the size class (in mm). Finally, to obtain the dry weight of the particles, each particle was carefully cleaned under de-ionized water and dried (at 40 °C) until stable weight, which was obtained with a precision scales (Ohaus Explorer EX225D/AD ± 0.1 mg).

### Contamination prevention

Between all steps, the equipment was rinsed with filtered de-ionized water and 35% ethanol (0.2 μm membrane filter, Whatman). During work laboratory coats made of cotton were worn and all samples not in use were covered with either aluminium foil or glass ware. During field sampling, we first took the microplastic samples to avoid influences of our own presence on the field, regarding microplastic contamination. One negative control was initiated during sampling and underwent all laboratory procedures.

## Data Availability

All data needed to evaluate the conclusions in the paper are present in the paper. Additional data related to this paper may be requested from the authors.
